# Dynamic Changes in Mucus Thickness and Ion Secretion during *Citrobacter rodentium* Infection and Clearance

**DOI:** 10.1371/journal.pone.0084430

**Published:** 2013-12-30

**Authors:** Jenny K. Gustafsson, Nazanin Navabi, Ana M. Rodriguez-Piñeiro, Ala H. A. Alomran, Pushpa Premaratne, Harvey R. Fernandez, Debashish Banerjee, Henrik Sjövall, Gunnar C. Hansson, Sara K. Lindén

**Affiliations:** 1 Department of Medical Biochemistry and Cell Biology, Sahlgrenska Academy, University of Gothenburg, Gothenburg, Sweden; 2 Department of Internal Medicine, Sahlgrenska Academy, University of Gothenburg, Gothenburg, Sweden; University of Kansas School of Medicine, United States of America

## Abstract

*Citrobacter rodentium* is an attaching and effacing pathogen used as a murine model for enteropathogenic *Escherichia coli.* The mucus layers are a complex matrix of molecules, and mucus swelling, hydration and permeability are affected by many factors, including ion composition. Here, we used the *C. rodentium* model to investigate mucus dynamics during infection. By measuring the mucus layer thickness in tissue explants during infection, we demonstrated that the thickness changes dynamically during the course of infection and that its thickest stage coincides with the start of a decrease of bacterial density at day 14 after infection. Although quantitative PCR analysis demonstrated that mucin mRNA increases during early infection, the increased mucus layer thickness late in infection was not explained by increased mRNA levels. Proteomic analysis of mucus did not demonstrate the appearance of additional mucins, but revealed an increased number of proteins involved in defense responses. Ussing chamber-based electrical measurements demonstrated that ion secretion was dynamically altered during the infection phases. Furthermore, the bicarbonate ion channel Bestrophin-2 mRNA nominally increased, whereas the Cftr mRNA decreased during the late infection clearance phase. Microscopy of Muc2 immunostained tissues suggested that the inner striated mucus layer present in the healthy colon was scarce during the time point of most severe infection (10 days post infection), but then expanded, albeit with a less structured appearance, during the expulsion phase. Together with previously published literature, the data implies a model for clearance where a change in secretion allows reformation of the mucus layer, displacing the pathogen to the outer mucus layer, where it is then outcompeted by the returning commensal flora. In conclusion, mucus and ion secretion are dynamically altered during the *C. rodentium* infection cycle.

## Introduction

Mucus is the first barrier a pathogen encounters when entering the human body [1]. In the colon, mucus consists of two layers: an inner sterile adherent mucus layer which is physically difficult to dislodge, and a thicker, loose, easily removed, outer mucus layer, which is the habitat of the commensal flora [2], [3]. This extracellular mucus barrier is comprised of an enormous net-like scaffold provided by the secreted polymeric Muc2 mucin [4]. This mucus contains both non-specific and specific anti-microbial proteins such as immunoglobulins and a number of other proteins with largely unknown function [4]. In addition to the luminal mucus with its gel-forming Muc2, the intestinal enterocytes also expresses a range of cell surface mucins [1].

*Citrobacter rodentium* is a member of a group of pathogens that colonize the lumen of the host gastrointestinal tract via attaching and effacing lesion formation. *C. rodentium* is used as a murine *in vivo* model system for the clinically significant diarrhea caused by attaching and effacing enteropathogenic *Escherichia coli,* as this pathogen does not cause disease resembling the human infection in mice. Several pathogens have been shown to interact with mucins, including enteropathogenic *E. coli* and enterohemorrhaghic *E. coli* which bind to bovine mucins, and *C. rodentium* which binds to murine Muc2 [5]–[8]. In contrast to wild type mice, which clear the infection spontaneously, 90% of *C. rodentium* infected mice lacking the Muc2 mucin succumb to the infection before day 8 [9]. These authors also showed that *C. rodentium* could be present in close association with the epithelial cells under the inner mucus layer. High numbers of *C. rodentium* were found in secreted Muc2 in infected animals *in vivo,* indicating that mucins may limit bacterial access to the epithelial surface [6]. During *C. rodentium* infection, the highest bacterial density and the highest colitis scores are found in the mid- and distal colon, whereas the parts of the intestine proximal to the mid colon are largely unaffected [6]. Using immunohistochemistry, we previously showed that mid- and distal intestinal expression of mucins (Alcian blue/PAS, Muc1, Muc2, Muc4, Muc13 and Muc3/17) differed between healthy and *C. rodentium*-infected mice [6].

B cell deficient mice infected with *C. rodentium* develop severe pathology in the colon and internal organs, fail to clear the infection and deteriorate rapidly [10]. However, only 50% of wild type mice have generated a *C. rodentium* specific immunoglobulin response by the time when the *C. rodentium* density starts to decrease, indicating that the immunoglobulins *per se* are not responsible for the decrease in bacterial density [6]. Recently, it was shown that germ-free mice, in spite of mounting a similar inflammatory response, do not clear the infection, and that adding the commensal flora at day 21 post infection, cleared the infection [11]. Germ-free mice have a very thin, disorganized mucus layer [2]. Microbial products and inflammatory cytokines stimulate increased production of mucins by mucosal epithelial cells, which cause a massive discharge of mucin in response to stimuli [12]. Stimulated mucin release occurs rapidly and is accompanied by hydration, resulting in an approximately thousand-fold expansion in volume [13], [14]. To understand and define how mucus protects epithelial surfaces is a challenge because of the complexities of mucin molecules and the numerous events associated with their secretion. Mucus swelling and hydration are strongly influenced by pH and ionic strength, i.e. by the composition of the luminal electrolyte and fluid environment, which depends on epithelial ion transport [13], [15], [16].

The aim of this study was to investigate the mucus dynamics, as well as goblet cell and enterocyte function during infection and clearance in the self-limiting *C. rodentium* infection model. We analysed a number of aspects assumed to be important for mucus formation: mucus thickness, mRNA levels of mucins and ion channels, estimated mucin amounts, basal and stimulated electrogenic properties, and changes in the mucus proteome. We found that mucus secretion and ion channel transport dynamically changes during the infection cycle. The increased mucus thickness during clearance, appear to be due to an altered secretory response.

## Results

### The Thickness of the Mucus Layer is First Reduced and then Increases, Reaching its Maximum during the Infection Clearance Phase

**Figure 1:**
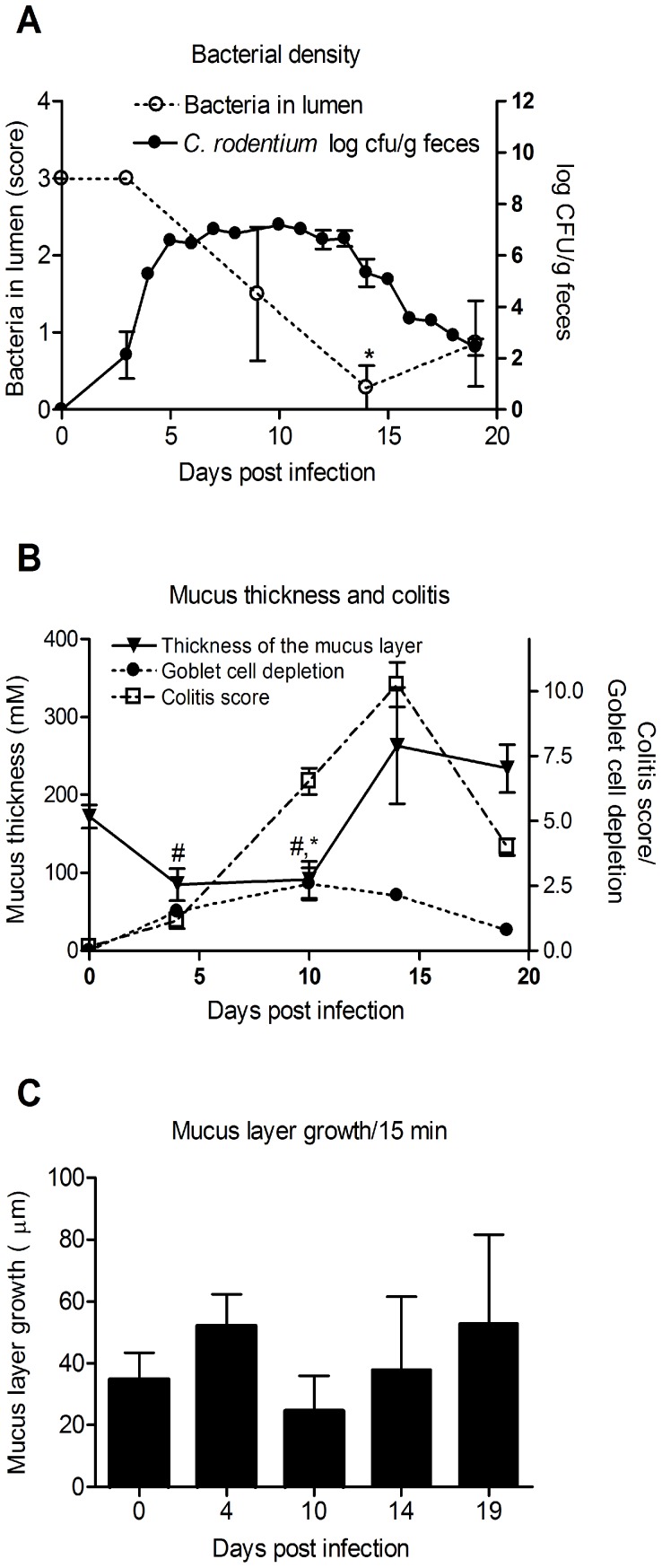
*C. rodentium* density, colitis score and mucus thickness and growth during infection. A: Colony forming units (CFU) of *C. rodentium* were analyzed in fecal pellets collected from individual mice. Compared to non-infected, all time points had an increased amount of *C. rodentium* (p<0.05). The total amount of luminal bacteria in the distal colon was scored in DAPI stained sections: Score 3 = same density as non-infected animals, 2 = medium density, 1 = low density and 0 = no bacteria detected (*p<0.05). Statistics: ANOVA, Dunnet’s post hoc test. B: The thickness of mucus layer changed during infection with lowest thickness between day 4 to 10 and the highest at day 14 post infection, whereas goblet cells depletion and colitis increased to the highest score at day 10 and 14 post infection, respectively. C: The thickness of the inner mucus layer changed during the course of infection, and reached its highest thickness at the start of the decrease in fecal *C. rodentium* density (CFU/gram feces). The distal colon explant was mounted and the thickness measured with a scaled micropipette, p<0.05 # vs day 14 and 19, * vs day 0. C: The *ex vivo* growth of the adherent mucus layer measured for the first 15 minutes after mounting the distal colon tissue. No significant differences were observed between the different time points (n = 5–12). Statistics: ANOVA, Tukey’s post hoc test.

In *C. rodentium* infected animals, measurement of bacterial density in feces revealed that in most mice the highest bacterial load occurred around day 10 and that the infection was cleared (i.e. less than 100 CFU *C. rodentium*/g feces) around day 19 (Figure 1A). The clearance of *C. rodentium* occurred gradually with a drop in CFU of 1–2 logs each day in most mice, although a 3 log CFU drop was found in 25% of the mice within a 24 h period. Thus, the clearance takes several days from start to finish. We chose four different time points reflecting the different components of the infection: day 4 (early infection), day 10 (highest fecal *C. rodentium* density), day 14 (decrease of bacterial density) and day 19 (cleared infection). The total amount of luminal bacteria in the distal colon, including the commensal flora, decreased during the infection, reaching its lowest point at day 14 (Figure 1A), which is partially explained by that only little fecal material was found in the distal colon at this time point.

Distal colonic explant tissue from these time points during the infection were mounted in a horizontal Ussing-type chamber and the mucus thickness and growth were monitored as previously described [17]. The inner adherent mucus layer thickness decreased during the early and mid- infection time points (4 and 10 days post infection), but then increased by day 14 and remained increased throughout day 19 (Figure 1B). The most severe inflammation, assessed as colitis score (crypt architecture, increased crypt length, emptied goblet cells, leukocyte infiltration, presence of lamina propria neutrophils, crypt abscesses, and epithelial damage and ulceration) was observed at day 14, at the time when the bacterial counts started to decrease (Figure 1B). Despite the observed changes in thickness of the adherent mucus layer that occurred during infection, the *ex vivo* thickness growth per 15 minutes of the combined inner and outer mucus layers did not differ significantly between time points (Figure 1C).

### Mucin mRNA Levels Increase during Early Infection

**Figure 2:**
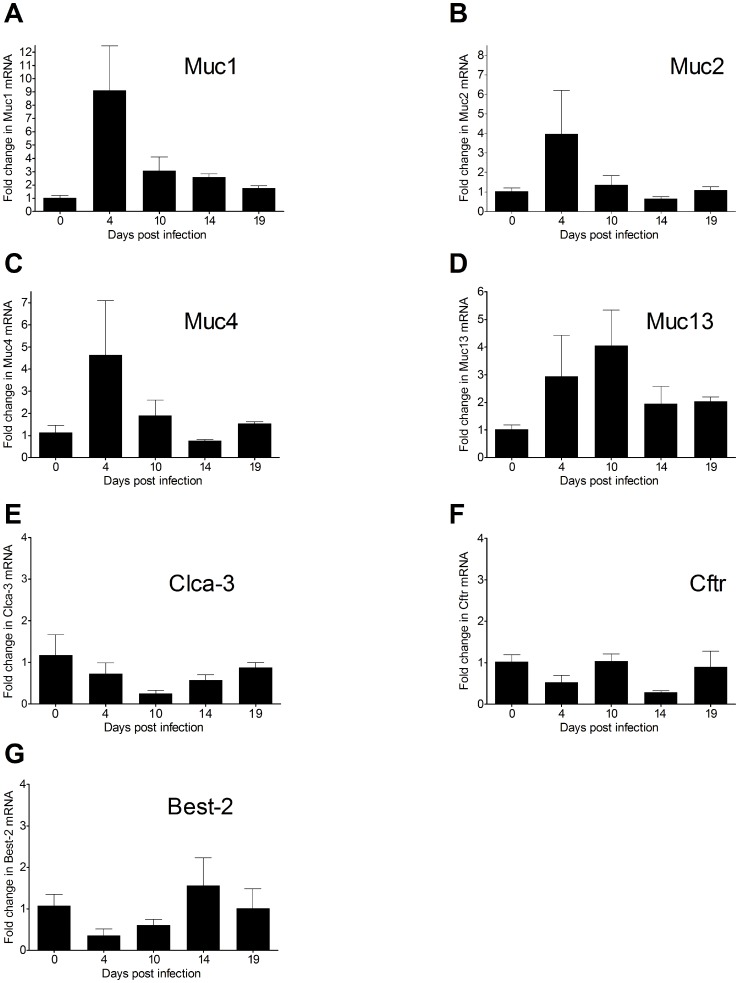
mRNA levels of mucins and some related proteins in distal colon during *C. rodentium* infection. Muc1 (A), Muc2 (B), Muc4 (C), Muc13 (D), Clca-3 (E), Cftr (F), and Best-2 (G). Expression data were normalized against the Hprt-1 housekeeping gene. Fold changes were calculated using ΔΔCT with mean of CT from three uninfected mice as control.

The mRNA levels of the Muc1, 2, 4 and 13 mucins were all increased (4–9 fold) during the earliest time points of infection (Figure 2). Muc6 and Muc5ac were not expressed in the colon at any time point (data not shown). Clca-3 was used as a goblet cell marker, and in line with our previously published results showing loss of goblet cells into the colonic lumen 12 days post *C. rodentium* infection [6], Clca-3 mRNA was decreased at day 10 (Figure 2F). During the bacterial expulsion and recovery phases (days 14 and 19) when the mucus layer was thicker, the mucin mRNA levels were similar to pre-infection levels (compare Figure 2 with Figure 1). Thus there is a 10 day difference between the increased mRNA and the increased mucus thickness and Muc2 protein levels, suggesting that mucus thickness and Muc2 protein level is controlled by other mechanisms than mucin mRNA levels.

### The Amount of Stored Mucins Change during the Infection Cycle

**Figure 3:**
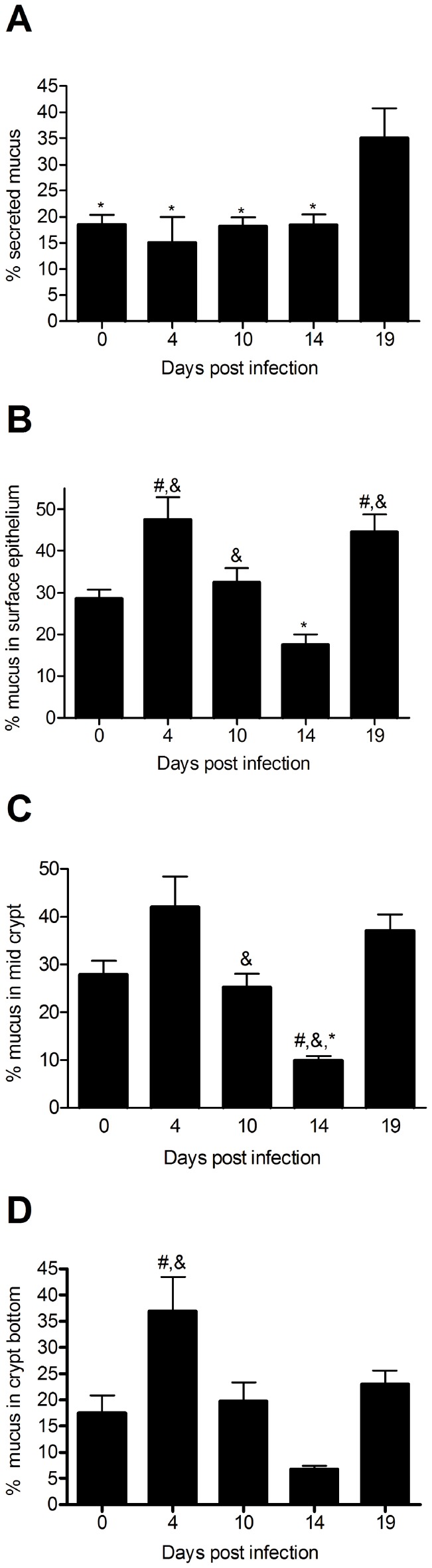
Quantification of total mucus as determined by Alcian blue/PAS stain in the distal colon. The percentage of the lumen (A) surface epithelium (B), mid-crypt (C) and bottom of the crypt (D) that was Alcian blue/PAS positive was calculated using the Image J image analysis program, n = 7–12, statistics: ANOVA, p<0.05, # vs day 0, & vs day 14, * vs day 19.

To determine whether the mucus production and secretion were more prominent in the surface or bottom of the crypts, we analyzed the Alcian blue/PAS staining (stains the carbohydrate moieties) of the tissue for the surface, mid- and bottom of the crypts. The alterations that occurred were similar at all three levels of the crypt (Figure 3B–D). The highest amount of mucin engorgement, i.e. the fullest goblet cells, was observed early during infection on day 4, which is consistent with the highest mRNA levels (compare Figure 2 with Figure 3). After day 4, the amount of mucin in the tissue decreased. At day 19 (after clearance) the amount of mucin in the tissue increased again and became higher than before infection (Figure 3B–D). Thus, it appears that during early infection, the mucin is accumulating in response to the infection and that the mucus depletion observed during the height of infection and clearance (days 10–14) is likely due to increased mucus secretion.

### The Electrophysiological Properties of the Colonic Epithelium Decrease during the Most Severe Colitis

**Figure 4:**
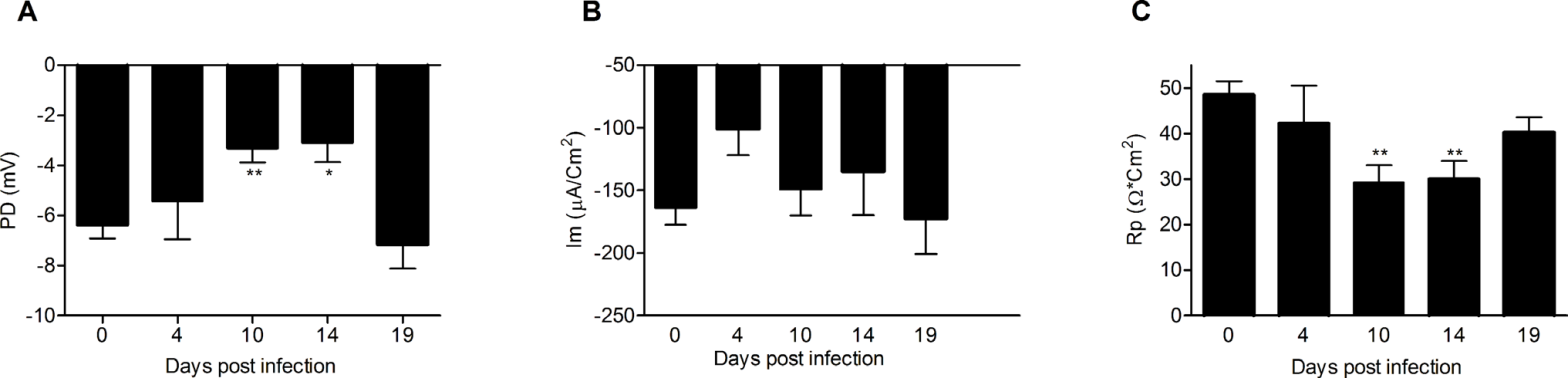
The basal electrochemical properties of the distal colonic explants measured in Ussing chambers during *C. rodentium* infection. Baseline PD (A), Im (B) and Rp (C) of distal colonic tissue during *C. rodentium* infection. Statistics: ANOVA, Dunnet’s post hoc test, * = p<0.05, ** = p<0.01.

The colonic explants mounted into Ussing chambers were analyzed by measuring transmucosal potential difference (PD), net membrane current (Im), and transepithelial resistance (Rp). PD and Im require functional active transport of ions across the epithelium and reflect sufficient ATP levels and ion channel activities in the epithelium, whereas Rp reflects opening and closing of epithelial ion channels and the electrical resistance of tight junctions. During the most severe colitis at day 10 and 14, PD and Rp both decreased (p<0.05, Figure 4). The magnitude of these parameters correlated weakly with the colitis score (p<0.001, Rp; Spearmans Rho = −0.365, PD; Spearmans Rho = −0.369). The decrease in Rp can at least be partially explained by an increase in paracellular permeability, as increased paracellular permeability previously has been demonstrated in *C. rodentium* infected C57Bl6 mice [18]. No significant changes in baseline Im were seen during the infection. The observation that basal net ion transport (Im) was not affected during the course of infection does not necessarily mean that epithelial transport was unaffected by the infection, only that the balance between basal rates of electrogenic absorption and secretion was unchanged.

### The Epithelial Response to Induced Secretory Stimuli Decreased Reversibly during Infection

During infection and inflammation, enteric pathogens are known to induce mucus and fluid secretion which coincide with ion secretion. This may contribute to mucus hydration and wash away pathogens. The relative importance of chloride and bicarbonate in these processes is not fully understood, but we recently showed that bicarbonate is important for formation of a normal mucus layer in the ileum as it was able to expand the stored Muc2 mucin at secretion [19]. How this transpires in the colon is not understood, but a colon specific HCO_3_^–^ ion channel, Bestrophin-2, was recently shown in colon goblet cells [20]. Murine colonic Ca^2+^ activated anion secretion is a biphasic event including a rapid Cl^−^ dependent secretion and a slower sustained HCO_3_^–^ dependent secretion [20]. The chloride transporting channel, the cystic fibrosis transmembrane conductance regulator (Cftr) is located to the apical enterocyte surface and is activated by cyclic AMP (cAMP) [21], but probably has a less important role during normal homeostasis in the colon than in the small intestine as its absence does not cause any disease symptoms [21].

To confirm the specificity of the secretagogues forskolin (cAMP) and carbachol (Ca^2+^) in the activation of these pathways, we first determined the response of the enterocyte-like cell line Caco-2 and the goblet cell-like cell line, LS513. As shown in our previous publication [22], stimulation with forskolin induced a secretory response in both Caco-2 and LS513 (Table 1). In contrast, stimulation with carbachol, only induced a secretory response in LS513 (Table 1). The presence of the CFTR inhibitor GlyH-101 inhibited the secretory response to forskolin in both the Caco-2 and LS513 cells whereas the response to carbachol was unaffected by the CFTR inhibitor (Table 1). Thus, the results suggest that forskolin and carbachol activate different pathways. We therefore used forskolin (cAMP mediated) and carbachol (Ca^2+^ mediated) to study anion secretion during murine *C. rodentium* infection.

**Table 1:** Change in membrane current (ΔIm) after stimulation with forskolin and carbachol with and without pre-treatment with the CFTR inhibitor GlyH-101.

Cell line	Caco-2	LS513
Forskolin ΔIm (µA/cm^2^)	−14±0.7	−323±53
% inhibition of forskolin ΔImby GlyH-101	90%	75%
Carbachol ΔIm (µA/cm^2^)	−0,127±0,14	−8.4±1.0
% inhibition of carbachol ΔImby GlyH-101	Not determined	0%

(mean±SEM, n = 6). As the Caco-2 cells did not respond to carbachol, the ability of GlyH-101 to inhibit the response was not analyzed in this cell line.

**Figure 5:**
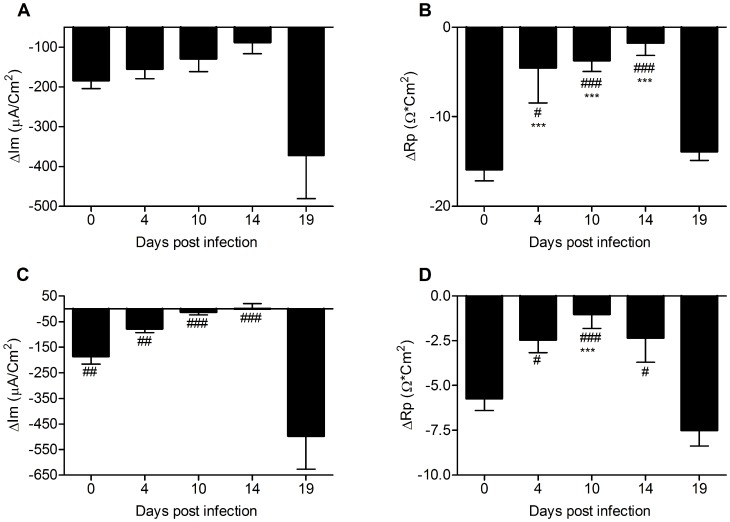
The response to forskolin and carbachol is altered during infection in distal colon explants studied in Ussing chambers. Changes in Im (A and C) and Rp (B and D) in response to forskolin (A–B) and carbachol (C–D) in distal colonic tissue during *C. rodentium* infection. Statistical analysis was performed on log transformed data: ANOVA Tukey’s post hoc test: p<0.05 #,*, p<0.01 ##,**p<0.001 ###,***, # vs day 19, * vs day 0).

During the course of *C. rodentium* infection, the ability of the murine colonic epithelium to mount a secretory response to both Ca^2+^ and cAMP mediated stimuli was altered (Figure 5). After clearance of the infection (day 19), the epithelial response to stimulus via both pathways was restored to pre-infection levels or enhanced. The Im response to forskolin did not change during the course of infection, whereas the Rp response decreased (Figure 5), which indicates that the ability to either open Cftr or basolateral K^+^ channels decreased during infection and inflammation. Similarly, the secretory response to carbachol gradually decreased during day 4, 10 and 14 post infection and was then increased at day 19 (Figure 5C). This suggests an increased fluid secretion during bacterial clearance, which would probably also increase the mucus turnover.

The ratio between the secretory responses to carbachol and forskolin was fairly constant during most time points of the infection course. However, at 14 days post infection (i.e. start of clearance), there appears to be distinct differences compared to the other time points: the relationship between the Im responses are changed, and there was a 6-fold change in the ratio between the change in Rp in response to carbachol (p<0.05) and similarly a 6-fold increase in the ratio between the nominally increased Bestrophin-2 and decreased Cftr mRNA (p<0.05). These data suggest that the ionic environment in the colon secretion and mucus was different at this time point.

**Figure 6:**
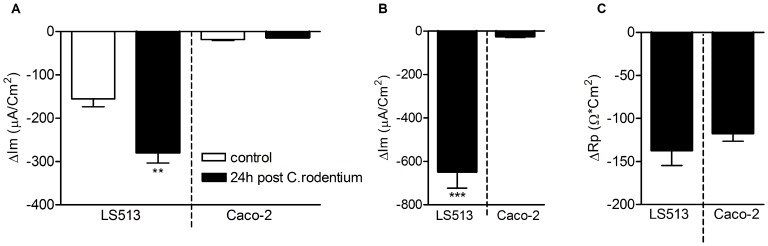
Ussing chamber responses are larger in a goblet cell-like *in vitro* model than in an enterocyte-like model after infection with *C. rodentium*. A: Membrane current (Im) response to forskolin of the goblet cell containing model (LS513) *vs* enterocyte like model (Caco-2) co-cultured with *C. rodentium* for 24 h (black bars) and cells without bacteria (white bars). B: Membrane current (Im) response of LS513 vs Caco-2 cells treated with *C. rodentium* for 30 min after insertion into the Ussing chamber and then stimulated with carbachol and forskolin. C: Transepithelial resistance (Rp) response of LS513 vs Caco-2 cells treated with *C. rodentium* for 30 min after insertion into the Ussing chamber and then stimulated with carbachol and forskolin. (2-tailed T-test, **p<0.01, ***p<0.001).

The *in vitro* secretory response to both forskolin and carbachol was enhanced in the goblet- cell containing cell line model LS513 after co-culture with *C. rodentium*, but not in the enterocyte like Caco-2 model (Figure 6). Thus, in short term infections (30 min - 24 h), the secretory responses appear enhanced in a goblet cell regulated manner. In the *in vivo* experiments, the only time we see an increase in secretory responses compared to non-infected mice is at the 19 day post infection time point, i.e. when the numbers of *C. rodentium* are very low and the inflammation is decreasing, indicating that factors in the surrounding tissue has a major impact on regulating these responses.

### Mucus Alterations during Infection

**Figure 7:**
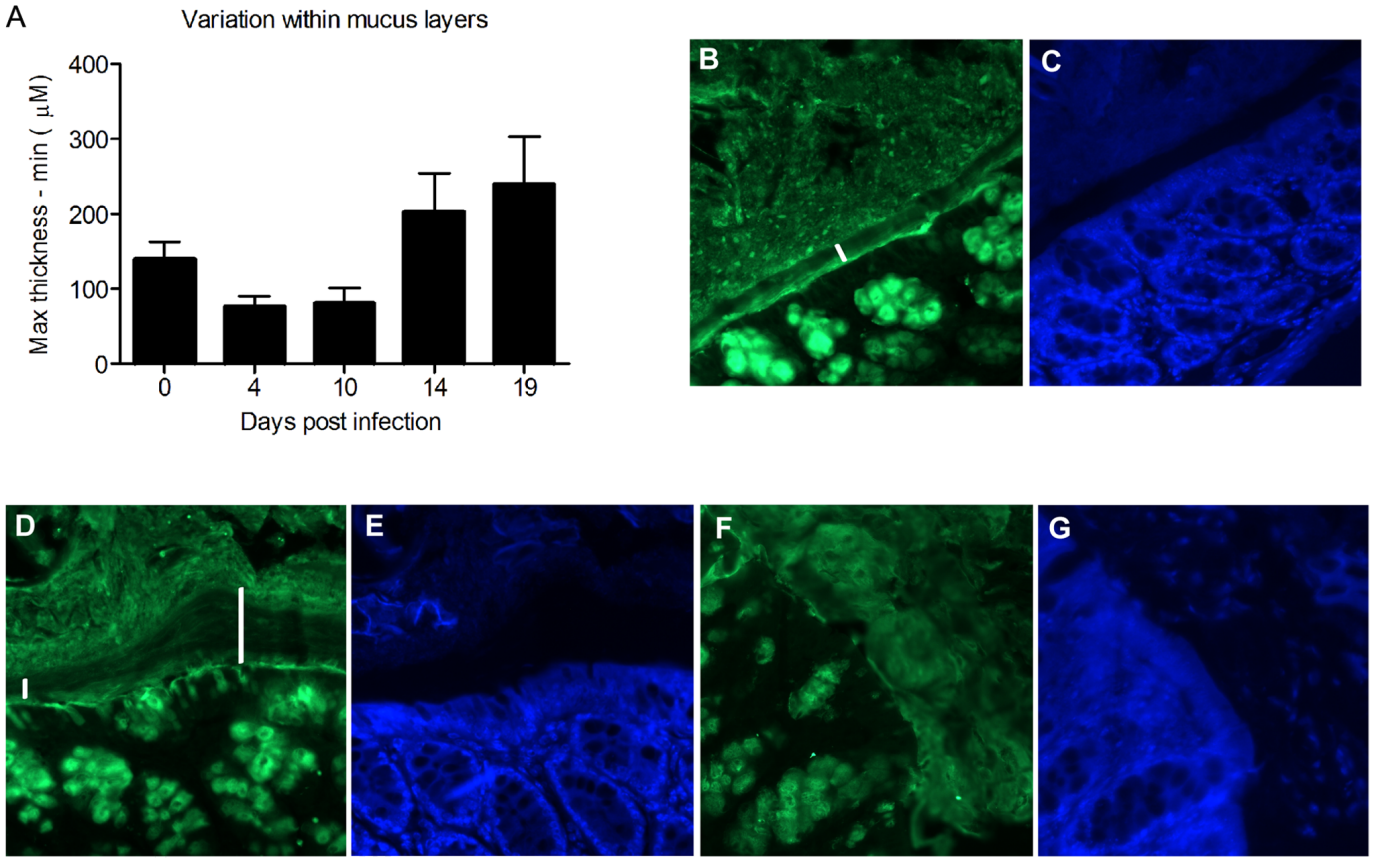
Increased variability in mucus layer thickness during clearance. A: The variation in thickness (i.e. the range, as measured with a micropipette in tissue explants) within each sample was altered at the different time points of infection (p<0.05, ANOVA F-test). B: Muc2 (green) stained distal colon of a non-infected C57BL/6 mouse. The inner, highly organized Muc2 layer is indicated by the white bar. C: The section from panel B stained with DAPI. The inner mucus layer is visible as a non-stained black band. D: Muc2 stained distal colon of a mouse infected for 19 days with *C. rodentium*. The striated adherent inner Muc2 layer is indicated by white bars; one bar has been placed in an area where the inner mucus layer appears thin, and the other one in an area where it is thick. E: The section from panel D stained with DAPI. F: Muc2 stained distal colon of another mouse infected for 19 days with *C. rodentium*. The adherent Muc2 layer is disorganized. G: The section from panel F stained with DAPI. Sloughed off cells are visible within the inner mucus layer.

**Figure 8:**
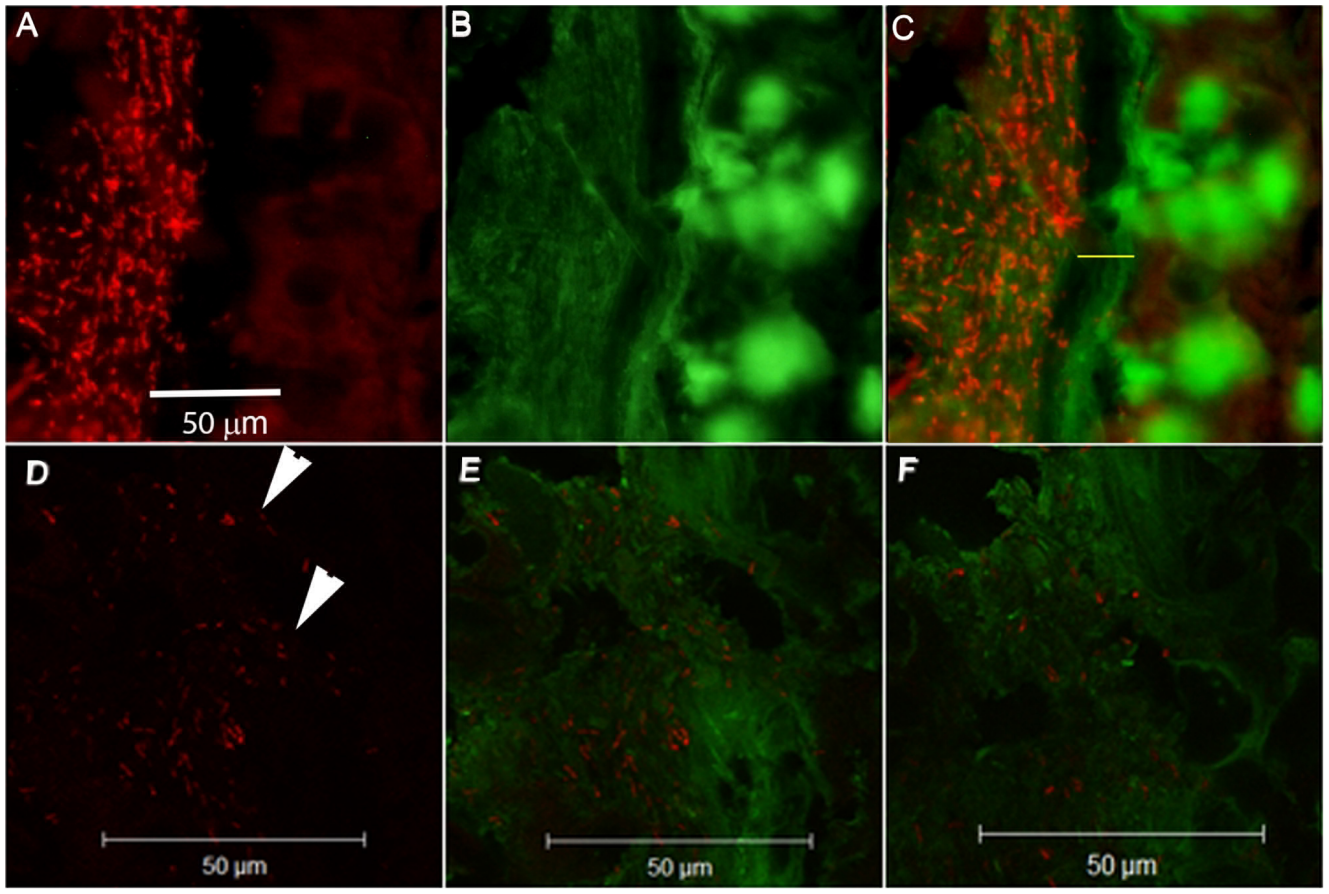
Mucus and bacteria during bacterial expulsion (day 14). A–C: The anti-Muc2C3 immunostained Muc2 mucin (green) formed an inner mucus layer (marked with a yellow bar in panel C) that is less well organized than in uninfected colon. Bacteria (FISH, eubacterial probe, hybridizing with both *C. rodentium* and other eubacteria) are mainly present in the outer mucus layer. Photos were taken with a Nikon Eclipse 90 i microscope, white bar = 50 µm. D–F: The mucus stained as in A–C showing the rod-shaped bacteria within the green mucus (white arrows), white bar = 50 µm. Photos taken with a LSM 510 META (Zeiss) confocal microscope with a 40x objective.

The variation in thickness as measured with a scaled micropipette in tissue explants within each animal was highest during the clearance phase (day 14 and 19 post infection, Figure 7A). In normal wild-type mice, the inner mucus layer is highly organized, devoid of bacteria and has a higher Muc2 density than the outer loose mucus layer [4]. This was also seen in the *C. rodentium* infected mice at day 0 and 4, as well as after clearing the infection (day 19, Figure 7). At day 19 post infection, the striated mucus layer appeared thick, but also highly variable in thickness (Figure 7D–G), consistent with the increased thickness and variability in thickness measured in explants (Figure 1A and 7B). In some mice, large amounts of sloughed off cells were still evident in the mucus layer at day 19, indicating that regenerative processes are still ongoing (Figure 7G). At day 10, an organized striated inner mucus layer was rarely found, and at day 14 it appeared present in some mice but not in others, indicating that a transition occurs around day 14. At this time point, the inner striated mucus layer, when present, was less organized (Figure 8). However, it appears that the mucus barrier is still functional, since very few bacteria were found in close vicinity of epithelial cells, and the amount of bacteria in the lumen was decreased at day 14 (Figure 1A). In most mice, no bacteria were detected in the lumen (except in the fecal material) or on the epithelial surface or in crypts at the 14 day time point (Figure 1A). The bacteria detected at the 14 day time point were present in fecal material, although very rarely (in one of 6 mice) bacteria could be detected close to the epithelial surface (data not shown). These data are in line with previously published results from the time point 6 days post infection, that showed that *C. rodentium* can cross the mucus layer and be present in close proximity to the epithelial cells [9], although our results show that this is a much rarer event during the clearance phase. When bacteria were present in the mucus, confocal microscopy revealed that the bacteria were present in close contact with the mucus (Figure 8D–F), in line with that Muc2 binds to *C. rodentium* [6].

### Proteomic Studies during the Clearance Phase Show an Increased Level of Proteins Belonging to Defense Response Pathways and Ion Channel Complexes

The increased mucus thickness could theoretically be a result of the inflammatory process and leakage of proteins such as fibrin. However, Massons Trichrome stain did not show any evidence of fibrin in the mucus layer (data not shown). To further address this and the question of other protein differences explaining the altered mucus, proteomic analysis of mucus collected from explants of four mice after 14 days of infection were compared to four uninfected controls. Proteins previously known to be associated with murine colonic mucus [23] were largely unaffected, no fibrin peptides were detected and other proteins that could confer mucus-like properties were also absent. A qualitative analysis of the mucus composition revealed several proteins that appeared mostly or only in infected mice (Table S1). For example the calcium pump Atp2a2 appeared in most of the infected samples and only in one control. Three proteins had significantly different levels in uninfected compared to infected mice (*p*<0.05): carbonic anhydrase 2 (Car2 decreased (3.7 times); farnesyl pyrophosphate synthase (Fdps; Q920E5, increased 3.2 times) and MYG1 (Myg1, not found in infected mice). Several proteins expected to be present in the epithelial cells appeared in the mucus of infected mice and is likely due to shed cells. Analysis of the proteomics data for gene ontology (GO) terms showed an increased number of proteins involved in defense responses in the infected mice compared with non-infected, while at the same time proteins related to cell differentiation and cytoskeletal processes were decreased (not shown, Table S1). Proteins annotated as “ion channel complexes” also appeared significantly more times in the proteins found in infected mucus than in controls, in line with the altered secretion observed during the clearance of the infection.

## Discussion

Here we demonstrated that during the self limiting *C. rodentium* infection, mucin expression, secretion and thickness dynamically change, and an increased thickness and less organized mucus coincide with removing *C. rodentium* and a large proportion of the commensal flora from the colon. Alterations in mucus properties such as thickness and organization depend on a number of factors such as rate of biosynthesis, exocytosis, and expansion/hydration. During the course of infection we observed a transient change in mucus thickness characterized by an initial decrease that shifted towards a thicker mucus layer during the clearance phase. The observation that this thickening coincides with the onset of clearance suggests that the mucus is involved in removing the pathogen; indeed the important role that mucus secretion plays in the removal of pathogens has previously been described for nematode infections [24], [25]. In murine worm infections, the Th1 vs Th2 type immune responses can be modeled by using different worm infection doses, and in this model it was shown that Th2 type cytokines, but not Th1, are involved in induction of goblet cell proliferation and mucin glycoprotein synthesis in the intestine [24]–[26]. The expulsion of *Nippostrongylys brasiliensis* and *Trichuris muris* is preceded by goblet cell hyperplasia and increased mucus secretion [24]–[26]. In *T. murins* infection, *de novo* synthesis of Muc5ac seems crucial for clearance of the worm, and the Th2 cytokine IL-13 appears important for the induction of Muc5ac [24]. *C. rodentium* induce a Th1/Th17 type response [27], and in line with this, we detected no Muc5ac mRNA. In contrast to clearance of nematode infection, onset of clearance of *C. rodentium* was preceded by an early increase in Muc1, Muc2, Muc4 and Muc13 mucin transcript levels that had returned to pre-infection levels at least four days prior to clearance. This suggests a slow biosynthesis of mucins, which is supported by their large size and complexity. However, even more important is the fact that mucins, especially Muc2, have been shown to have significant posttranscriptional regulation. For example, in the small intestine of cystic fibrosis mice there is a 50% decrease in mRNA levels concomitant with the protein increasing 3-fold [28], [29].

During the expulsion phase of the *C. rodentium* infection, the inner colonic mucus layer was less organized and its stratified organization less apparent. Upon secretion the mucin molecules expand in volume >1,000 times, and the efficiency of this expansion process has a huge effect on the properties of the secreted mucus [30], [31]. Within the secretory granules the mucin molecules are stored at low pH and high Ca^2+^ conditions. During the exocytosis process Ca^2+^ has to be removed to allow for proper expansion of the molecule. This process is highly dependent on the ionic milieu that the protein is secreted into, which in turn is regulated by the transport function of the colonic epithelium [13]. Noticeably, one of the proteins we found predominantly present in infected mucus was the calcium pump Atp2a2, which could be involved in mucus production in an analogous manner to what has been shown for the synthesis of collagen, which similarly to Muc2, is a large molecule [32]. We recently showed that loss of Cftr mediated bicarbonate transport results in dense adherent mucus in the small intestine [15], [19], [33]. Our results from the infected mice showed that the responsiveness to both forskolin (cAMP) and carbachol (Ca^2+^) induced anion secretion was severely affected by the infection (in concordance with a previous study at 10 days post infection [34]) and the response was almost abolished during the early clearance phase. Carbachol induced Ca^2+^ mediated anion secretion in colon was recently shown to be dependent on the goblet cells specific transporter Bestrophin-2, whereas cAMP mediated secretion to a large extent is mediated via the enterocyte specific Cftr channel [15], [16]. Interestingly, the carbachol response was more severely affected compared to the forskolin response, suggesting that the infection and inflammation affect the goblet cells to a larger extent than the enterocytes. This is further supported by a study in Swiss-Webster mice that even showed an up-regulation of Cftr mediated secretion during infection [35].

In contrast to the observed loss of anion secretion in the infected mouse colon, *in vitro* infection of the goblet cell containing cell line LS513 in the absence of the inflammatory milieu resulted in an enhanced secretory response to both secretagogues. Thus, the effects on epithelial cells in response to the pathogen appear to be distinctly different from effects from the host inflammatory response. A possible explanation could be that exposure to *C. rodentium* has a direct effect on the epithelial cells, resulting in increased anion secretion, which is a previously characterized response to *V. cholera* [36]. However, in the intact colonic tissue, activation of the immune system and induction of an inflammatory response will likely harm the transport machinery. In both mouse and human colon, acute inflammation is associated with impaired absorptive and secretory functions, which among other things have been attributed to inhibition of the basolateral Na^+^/K^+^-ATPase which is the engine behind both absorption and secretion [37]. Further support for the effect of the bacteria on regulation of epithelial transport could be found in the proteomic analysis of the mucus, where we observed an increase in the ion channels and immune response-related proteins in mucus from infected animals. Furthermore, we could observe particular cases of inflammatory proteins more predominant in mucus at day 14 post-infection. This was the case for Ifi35, a protein that has been previously found induced by interferon in a pathway of antiviral defense [38]. Thus, it appears that the colonic epithelium responds to the infection by upregulating proteins involved in ion transport during the expulsion.

The ability to perform electrogenic anion secretion in response to additional stimuli does not appear to be crucial for clearance of the bacteria. However, the altered organization of the secreted mucus that were observed, and that appear to be the result of an altered balance between chloride and bicarbonate secretion, might be crucial to clear the infection. In support of the hypothesis that the colonic mucus is essential for clearing the infection, Bergström *et al.* recently showed that 90% of mice that are deficient in the Muc2 mucin succumb to the infection by day 8 post infection [9]. In this study the authors also analyzed changes in Muc2 expression in WT mice that showed a trend towards an increase in Muc2 mRNA expression at day 6 post infection. This correlates with our observations of an increase in Muc2 transcripts day 4 post infection. Although the initial increase in mucus mRNA was normalized, the decreased intracellular storage of mucins during the clearance suggest increased secretion, something that is also supported by the increased mucus thickness during this period. We have previously described the presence of large amounts of shed whole cells, including goblet cells in *C. rodentium*-infected specimens [6]. The factors causing premature death of the goblet cells with infection are unknown, although this can reflect an increased compound exocytosis, which may result in goblet cell death [39], [40].

We have previously demonstrated that Muc2 binds to *C. rodentium*, and the fact that we did not detect luminal bacteria (in most mice) during the early phase of clearance when the mucus layer reaches its thickest stage supports that mucus aids in flushing away the pathogen. This is further supported by previously published observations that the amount of luminal *C. rodentium* was 10–100 fold higher in Muc2 deficient mice, in spite of the growth stimulatory effect that Muc2 isolated from WT mice had on *C. rodentium* [9]. However, the inability of the Muc2 deficient mice to flush away *C. rodentium* may also be due to an impaired ion secretory response as a result of the more severe colitis, damaged epithelium, and increased bacterial density in these mice.

Quantitative protein changes were also observed for specific proteins. For instance, Myg1 showed a striking pattern, being completely undetected in infected samples. Previous studies pointed to a connection between this protein and stress and immune responses, mediated through still not totally understood functions in the mitochondria and/or nucleus [41]. Most interestingly, the carbonic anhydrase Car2 was decreased almost 4 times in mucus at day 14 post-infection, coinciding with the decreased response to carbachol, and a thick mucus layer. This enzyme is responsible for the reversible hydration of CO_2_ to H_2_CO_3_ [13]–[16], [22]. In the colon, the major pathway for constitutive bicarbonate secretion is via the apical Cl^−/^HCO_3_^−^ -exchanger downregulated in adenoma (Dra). Slc26a3 (Dra)-deficient mice display chloride-losing diarrhea, enhanced colonic proliferation, and distinct upregulation of ion transporters in the colon [42]. Bicarbonate secretion via Dra relies on intracellular production of bicarbonate via carbonic anhydrases including Car2, and studies have shown that inhibition of Car2 by azetasolamide reduces chloride dependent bicarbonate secretion by 75% in rat colon [43], [44]. In our case, a decreased activity of Car2 during infection would limit bicarbonate secretion via Dra, which in turn could affect secretion and/or expansion of the mucus during clearance of the infection.

In support of this hypothesis, our GO term analyses showed an increase in the ion channel complexes during day 14 post-infection, while the function termed as “regulation of body fluid levels” was decreased. Although little is known about which transport pathways that regulate mucus properties in the colon, a recent study showed that loss of the goblet cells specific sodium bicarbonate co-transporter NBCn1 results in a thinner adherent mucus layer and decreased mucus growth rates [45]. This together with our observation of a defective mucus layer in Slc93a^−/−^ mice that lack the sodium hydrogen exchanger 3 point toward both anion secretion and cation absorption being important for maintaining an intact mucus barrier [46].

**Figure 9:**
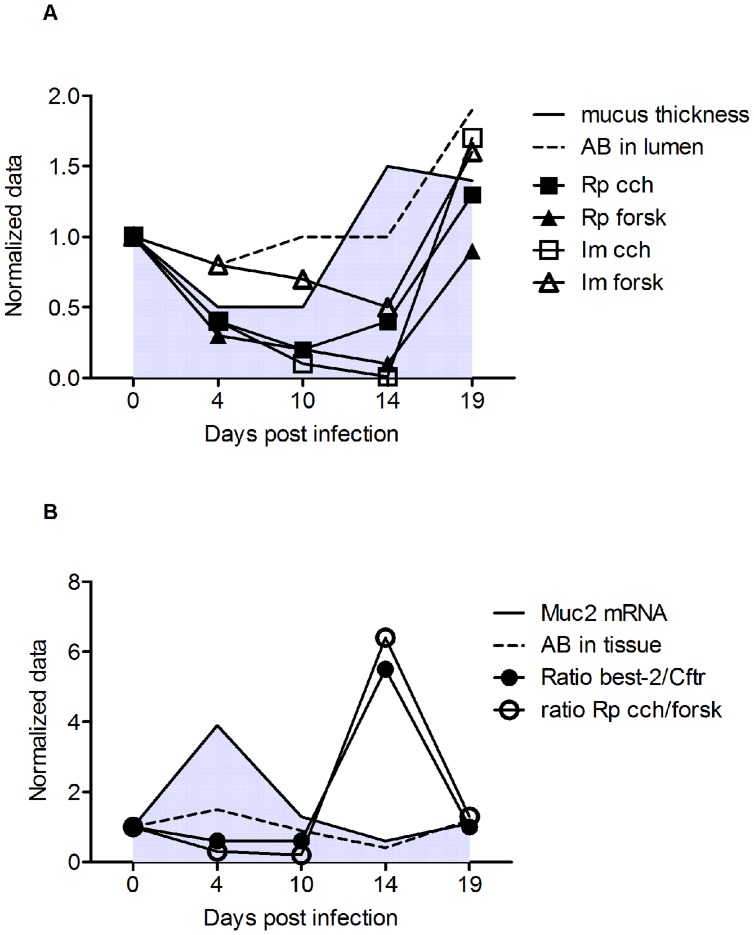
Summary of changes during the course the infection. The presented data are normalized against uninfected controls, which are set to 1. The actual data points and error bars have been presented previously in Figures 1–7. Panel A: Mucus thickness as measured with a scaled micropipette (grey filled area under a black line), mucin material present in lumen as measured with Alcian blue/PAS stain in fixed sections (––) and secretory responses to forskolin (Rp▴, Im Δ) and carbachol (Rp ▪, Im □). Panel B: Muc2 mRNA (grey filled area under a black line), mucin material present in tissue storage as measured with Alcian blue/PAS stain in fixed sections (––), ratio of Best-2 mRNA (•) and ratio of Rp in response to carbachol vs forskolin (○).

In summary, as illustrated in Figure 9, early during infection (day 4), the adherent mucus layer thickness and amount of luminal mucin material is decreased, but the amount of mucin in storage (Alcian blue in the tissue) is increased, together with an increase in mucin mRNA. At the same time, the ability of the tissue to mount a secretory response to forskolin and carbachol is decreased. At day 10, the mucin mRNA levels, mucin storage and amount of mucin in the lumen is similar to pre-infection levels, although the thickness of the adherent organized mucus layer is thinner. The secretory response to forskolin and carbachol remains decreased. At day 14, the adherent mucus layer is thicker but has a less structured appearance compared to the non-infected animals. Goblet cell depletion is still prominent, the mucin and goblet cell marker mRNA levels are similar to pre-infection levels, the level of Alcian blue positive material in the lumen are similar to pre-infection levels, and the amount of alcian blue positive material in the tissue is decreased, indicating that the mucus is regulated by other factors, such as the changed ionic composition indicated by Ussing experiments, ion channel mRNA and proteomics experiments. Together with the concurrent reformation of the striated mucus layer, these secretory changes appear to eliminate the bacteria from the close proximity of the epithelial cells and push them out to the outer mucus layer. Once outside the protective niche of the inner nominally sterile mucus layer, they can then eventually be outcompeted by the returning commensal flora, as recently shown [11]. At day 19 post infection, the mucus layer remains thicker, but the secretory responses are enhanced, whereas most other parameters have returned to similar levels as in the non-infected controls. The increase in both stored and luminal secreted mucus after infection may be a result of a decreased stimulus to secrete mucins, in combination with a decrease in mucus dissemination when the pathogen has disappeared and the normal microflora still has not recovered to pre-infection levels.

In conclusion, in the present study we show that during the self-limiting infection of *C. rodentium,* mucus transcription and secretion are dynamically altered in response to the infection and that clearance of the infection coincides with the reformation of the organized inner mucus layer and an increased mucus thickness. The increase in mucus thickness during *C. rodentium* clearance coincided with altered ion channel activities and together this could provide a partial mechanism for *C. rodentium* elimination from the rodent intestine.

## Methods

### Animals

6–8-week old, specific-pathogen-free, male C57BL/6 mice were purchased from Charles River (Germany). The mice were housed in individually ventilated cages at the Laboratory for Experimental Biomedicine (EBM) for the duration of the study. The animals had free access to water and food throughout the experiment and monitored daily.

### Ethics Statement

All experimental procedures were approved by the Göteborgs Djurförsöksetiska Nämnd (Ethic No. 261/09) based on the regulation from Djurskyddsförordningen DFS 2004∶4.

### Infection of Mice

*C. rodentium* strain ICC169 was grown on MacConkey agar (Oxoid, Hampshire, England) for 20 h at 37°C. Bacteria harvested from plate cultures were suspended in warmed Luria-Bertani broth to an OD_410_ of 8.00. Male C57BL/6 mice (age 8–12 weeks) were infected with 100 µl of the bacterial suspension containing 5×10^9^ colony forming units (CFU) by oral gavage. The inoculated culture was serially diluted and plated to confirm the CFU administered. Fecal samples were homogenized in broth, serially diluted, plated onto MacConkey agar, and grown for 20 h at 37°C. CFUs were enumerated by counting *C. rodentium* ∼1 mm diameter fuschia-coloured colonies. Uninfected control mice (n = 14) and mice at days 4 (n = 6), 10 (n = 15), 14 (n = 8) and 19 (n = 5) post infection, were anesthetized with isoflurane and killed by cervical dislocation. The infection experiments were performed on several occasions, and each time point contains results pooled from 2–5 experiments. The last 3 cm of colon, beginning at the anal verge, were collected. The most distal 2 cm colonic specimens were collected into ice cold transport KREBs buffer for immediate Ussing experiments and mucus growth measurements. The second distal 0.5 cm of colon were harvested into 0.5 ml fresh Carnoy’s methanol fixative (60% dry methanol, 30% chloroform, 10% glacial acetic acid) and the third distal 0.5 cm (40 ug) into 200 µl RNAlater (Ambion).

### Histological Assessment

For analysis of colitis, 7 µm sections of Carnoy’s fixed tissue were stained with haematoxylin/eosin, coded to blind the analysis, and the entire section was systematically scored: aberrant crypt architecture (0–3), increased crypt length (0–3), goblet cell depletion (0–3), general leukocyte infiltration (0–3), lamina propria neutrophil counts (0–3), crypt abscesses (0–3), and epithelial damage and ulceration (0–3) were scored. The colitis score presented in Figure 1 represents the sum of these individual scores.

### RT- PCR

0.5 cm of distal colon were collected in RNAlater™ (Ambion) and kept at 4°C over night, then at −80°C. Total RNA was extracted using the RNeasy mini kit (QIAGEN GmbH, Hilden, Germany) following the manufacturer’s protocol. QuantiTect Reverse Transcription kit (QIAGEN) was used for cDNA preparation from extracted RNA of each sample, according to the manufacturer’s protocol. SyberGreen based real time RT-PCR was carried out using primers for Muc1, Muc2, Muc4, Muc6, Muc13, Clca3 (sequence showed in Table 2) and Muc5AC, bestrophin-2 and Cftr (QIAGEN). Data were normalized with the Hptr-1 housekeeping gene from QIAGEN. The CT for Hptr-1 was consistent and did not change between time points of infection (less than one cycle difference). Fold changes were calculated using the ΔΔCT method with the mean of CT from three uninfected mice as control. Fold changes ≥4 were accepted as up-regulation and ≤0.25 as down regulation. Murine gastric tissue was used as a positive control for Muc1 and Muc5ac.

**Table 2:** List of primers for SyberGreen based RT-PCR.

	Primer	
Target	Forward	Reverse
Muc1	5′-GGTTGCTTTGGCTATCGTCTATTT-3′	5′-AAAGATGTCCAGCTGCCCATA-3′
Muc2	5′-GTCTGCCACCTCATCATGGA-3′	5′-CAGGCAAGCTTCATAGTAGTGCTT-3′
Muc4	5′-TCTTTCTGTCTCAACTGTTGAATCAGA-3′	5′-CGTGGCCAGGATGTCAAAC-3′
Muc6	5′-TGCTCCCAGAATGAGTACTTCGA-3′	5′-CAGAGGTGGAACTGTGAAACTCAGT-3′
Muc13	5′-GCCAGTCCTCCCACCACGGTA-3′	5′-CTGGGACCTGTGCTTCCACCG-3′
Clca3	5′-TTGAGCAGCTGTCCAAAATG-3′	5′-TGCGAAAGCATCAACAAGAC-3′

### Mucus Measurements

The colonic tissues used in the Ussing chamber experiments were divided into two parts, so that experiments were run in parallel for the electrophysiological data and the mucus thickness data. The mucus thickness experiments were performed as described previously [47]. Briefly, following incubation on ice for 30 min, the longitudinal muscle was removed and the tissue was mounted in the perfusion chamber. The apical and basolateral solution used in this set up were the same as described below for the Ussing chamber experiments. Tissue viability was measured using transepithelial potential difference recording (PD). To visualize the colonic mucus layer a suspension of activated charcoal particles was added to the apical surface and allowed to sediment down to the top of the mucus layer. The thickness of the mucus layer was assessed by measuring the distance between the top of the mucus layer and the epithelial surface using a micropipette (tip diameter, 5–10 µm) connected to a micromanipulator. The spontaneous mucus growth was measured every 15 min for 60 min.

### PAS/Alcian Blue Stain

De-waxed sections were immersed in 100% ethanol for 10 min, rinsed in water for 10 min, immersed in 3% acetic acid for 2 min and stained in 1% Alcian Blue 8 GX in 3% acetic acid (pH 2.5) for 2.5 h. Nonspecific stain was removed with 3% acetic acid and sections were rinsed in water for 10 min. The slides were then oxidized in 1% periodic acid in water at room temperature for 10 min, washed in water for 5 min, immersed in Schiff’s reagent for 10 min, rinsed in water for 5 min and then three times in 0.5% sodium meta-bisulphite before a final wash in water. ImageJ was used to quantify the percentage of tissue that was positive in the upper third, middle third and bottom third of the tissue by outlining these areas. ImageJ is a public domain image processing program (Wayne Rasband at the Research Services Branch (RSB) of the National Institute of Mental Health (NIMH), National Institutes of Health, Maryland, USA).

### Masson’s Trichrome Staining

Briefly, the Carnoy fixed intestinal tissue sections were re-fixed overnight at room temperature in Bouin’s fixative, followed by using the Masson’s Trichrome staining kit (Polysciences Inc., USA) according to the manufacturer’s instructions. The dyes employed during the staining procedure stained the collagen fibers blue, nuclei black and the connective tissue and muscle fibers red.

### Cell Culture

Caco-2 (derived from colorectal adenocarcinoma, ATCC) and LS513 (derived from colorectal adenocarcinoma, ATCC) cell lines were cultured in RPMI medium containing 15% and 10% fetal calf serum respectively, 2 mM L-glutamine, 100 units/ml penicillin G sodium and 100 µg/ml streptomycin. The cells were plated on snapwell tissue culture inserts (0.4 µm pores, 1.13 cm in diameter, Corning) at a density of 7.5×10^4^ cells/well. Both cell lines were cultured under standard conditions till complete confluency. The confluent Caco-2 cells were then cultured for another 21–24 days under standard conditions, whereas the LS514 were cultured in semi-wet interface for 21–24 days to allow the cells to differentiate as described previously [22]. For the 24 h infection experiments, *C. rodentium* was harvested into sterile PBS, and 10 µl of bacterial suspension with an OD of 2.0 at 410 nm was added to each well (Multiplicity Of Infection approximately 10) 24 h prior to mounting the cell cultures into the Ussing chambers. The same volume and concentration was added to the experiments where the co-culture time was 30 minutes, however, as the volume of the Ussing chamber was much larger (5 ml) the amount of bacteria reaching the cells was lower.

### Ussing Chamber Experiments on Cultured Colonocytes

The snapwell tissue culture inserts were mounted in vertical Ussing chambers (exposed area 1.13 cm^2^). The basolateral side of the membrane was immersed in KREB’s buffer (115.8 mM NaCl, 1.3 mM CaCl_2_, 3.6 mM KCl, 1.4 mM KH_2_PO_4_, 23.1 mM NaHCO_3,_ 1.2 mM MgSO_4,_ Merck, Darmstadt, Germany) containing 5.7 mM Na-Pyruvate, 5.1 mM Na-L-Glutamate and 10 mM D-Glucose, whereas the apical compartment was immersed in KREB’s buffer containing 5.7 mM Na-Pyruvate, 5.13 mM Na-L-Glutamate and 10 mM D-Mannitol. The solutions were gassed with 95% O_2_ and 5% CO_2_ at a temperature of 37°C and pH 7.4 throughout the whole experiment.

### Ussing Chamber Experiments on Mouse Colon

Following dissection, the tissue specimen was flushed with ice-cold KREB’s buffer to remove colonic content. The specimen was then kept on ice in KREB’s buffer for 30 min, opened along the mesenteric border and mounted in the Ussing chamber (exposed area 0.25 cm^2^). Both sides of the epithelium were bathed in 2 ml KREB’s buffer that was constantly gassed with 95% O_2_ and 5% CO_2_ at a temperature of 37°C and pH 7.4 throughout the whole experiment. The mucosal solution also contained 5.7 mM Na-Pyruvate (Sigma-Aldrich, Steinheim, Germany), 5.13 mM Na-L-Glutamate (Merck) and 10 mM D-Mannitol (BDH Laboratory supplies, Poole, England) and the serosal solution contained 5.7 mM Na-Pyruvate, 5.1 mM Na-L-Glutamate and 10 mM D-Glucose (Riedel-Haen AG, Hannover, Germany).

Transepithelial potential difference (PD) was measured once every minute during the whole experiment with a pair of matched Ag/Ag calomel electrodes (Radiometer, Copenhagen, Denmark) placed in saturated KCl and connected to the mucosal and serosal sides via a pair of 0.9% NaCl 6% agar bridges (bacteriological agar, Oxoid,). After mounting the tissue or the snapwell cell culture inserts in the Ussing chamber, PD was allowed to stabilize for 30 min to achieve steady-state conditions. Epithelial resistance (Rp) and net membrane current (Im) were measured using square-pulse analysis: 5 V, 3 ms pulses were generated by a square pulse generator (Medimet, Gothenburg, Sweden) via a current limiting resistor (138 kΩ for tissue culture samples and 98 kΩ for mouse tissue) connected to a platinum electrode and applied across the tissue sample. The mean voltage response curve from twenty measurements was calculated and a linear fit was applied to the mean graph resulting in the voltage at time zero. Rp and Im were assessed in 4.5 min intervals for the entire experiment. Samples were stimulated with 10 µM forskolin (Sigma-Aldrich, Darmstadt Germany) and/or 1 mM carbachol (Sigma-Aldrich, Darmstadt Germany). The CFTR inhibitor GLYH-101 (WVR, Sweden) was used at a concentration of 20 µM. The basal values presented in the figures are the mean values pre stimulation. Delta values are used to illustrate effects induced by the various substances used in the experiments (ΔPD, ΔRp and ΔIm) and were calculated by subtracting the baseline value from the maximum effect.

### Immunofluorescence

Paraffin embedded sections were deparaffinized and rehydrated. For antigen retrieval, sections were heated in 0.01 M citric acid buffer pH 6 for 30 min at 99°C. Non-specific binding was blocked by Protein Block (Dako, Copenhagen, Denmark) and the sections were stained with an antibody recognizing the Muc2 mucin (anti-MUC2C3 [2]) diluted (1∶500) in antibody diluent (Dako). Alexa Fluor 488-conjugated goat anti-rabbit immunoglobulins (Invitrogen, Eugene Oregon, USA) were used as secondary antibody. To visualize DNA, including bacteria, the sections were mounted with DAPI containing Prolong Gold antifade reagent (Invitrogen). Muc2 staining was evaluated by manual estimation of intensity and continuity of the mucus layer and positive cells. Sections were evaluated in a blinded fashion at 20x magnification Pictures were captured on an Eclipse 90 i microscope (Nikon, Tokyo, Japan) fluorescence microscope.

### In situ Hybridisation/MUC2 Immunofluorescence

The slides were deparaffinized, and then an abbreviated antigen retrieval was performed by placing them in 10 mM sodium citrate, pH 6.0 at 95°C for 10 minutes. The slides were briefly rinsed in distilled water, air dried, and hybridization solution (40% (v/v) formamide, 20 mM Tris-HCl pH 7.4, 0.9 M NaCl, 0.1% SDS) containing 10 ng/µl of Cy3.5 5′ labeled eubacteria-specific probe (5′-GCTGCCTCCCGTAGGAGT-3′) [48] added, and incubated at 37°C in a humidified chamber containing 40% formamide overnight. The following day, the slides were washed with 0.9 M NaCl, 20 mM Tris-HCl pH 7.4 at 50°C for 20 minutes, followed by a brief submersion in room temperature distilled water, and then blocked at 4°C in serum-free Protein Block (Dako) for 1 hour. The Muc2 immunohistochemistry was then carried out as above, with all steps done at 4°C: the primary antibody MUC2C3 was diluted 1∶500 and incubated overnight, washed with PBS containing 0.05% Tween 20 (PBS-T), then an Alexa-Fluor 488 conjugated anti-rabbit secondary antibody applied for 3 hours, washed in PBS-T and mounted. Pictures were captured on an LSM 510 META (Zeiss) confocal microscope with a 40x objective.

### Mass Spectrometry Analysis of Mucus Samples

Briefly, mucus from the distal colon of four uninfected mice and four mice infected with *C. rodentium* (14 days post infection) was collected under microscopy by gentle scraping in PBS supplemented with Complete EDTA-free protease inhibitor (Roche, Basel, Switzerland) and the samples were kept at −80°C. The collected mucus was prepared according to the FASP method [49]. The peptides obtained were then analyzed by nanoLC-ESI-MS/MS on a LTQ-Orbitrap XL (Thermo Scientific) using CID fragmentation as described before [50]. Full MS scans covered the 350–2000 m/z range, and MS/MS was performed for the eight most abundant multiply charged ions per scan. Peptides were excluded for 30 sec to allow lower abundance ions to be fragmented. Data were acquired and inspected with Xcalibur 2.0 (Thermo Scientific), and analyzed using MaxQuant 1.2.2.5 [51]. Data were searched with Andromeda [52] against the SwissProt mouse protein database (version 1203) and the standard MaxQuant contaminant database. The search was done allowing 2 missed cleavages for trypsin and 6 ppm of error for the main search. The modifications were set as fixed carbamidomethylation of Cys, and variable oxidation of methionines and acetylation of protein N-termini. The false discovery rate (FDR) was set to 0.01, and matching between runs through remapped retention time (2 min) was selected.

### Statistics

Statistical analyses were performed using the SPSS statistics 18 (IBM) or Prism (GraphPad Software, Inc.) programs. Due to the variation in spread of data between the groups for the mucus and Ussing chamber measurements, statistical analysis was performed on log transformed data. The remaining data sets were analyzed as non-transformed data. Comparisons of means were performed using ANOVA with either Dunnett’s post hoc test (to compare with the non-infected group) or with Tukey’s post hoc test (to make comparisons between all groups).

The proteomic results were analyzed in Perseus 1.3.0.4 (J. Cox, manuscript in preparation, software available at http://maxquant.org/index.htm). Protein quantities were calculated as intensities on the basis of the extracted ion current (XIC). For the quantitative comparison, we used a two-sided T-test with permutation-based FDR for truncation, 250 randomizations, and a threshold of 0.05. The presence or absence of proteins was studied through numerical Venn diagrams. The functional annotation and analysis of the protein sequences on the basis of the GO (Gene Ontology) terms was performed with Blast2GO [53].

## Supporting Information

Table S1 **Proteins found predominantly in the mucus of infected mice at day 14 (at least in 3/4 infected animals and in none or only 1/4 control mice), or almost absent from them (present in at least 3/4 control mice, but in none or only 1/4 infected mice).**
(DOCX)

## References

[1] McGuckin MA, Linden SK, Sutton P, Florin TH (2011) Mucin dynamics and enteric pathogens. Nat Rev Microbiol 9: 265–278. 21407243 10.1038/nrmicro2538

[2] Johansson ME, Phillipson M, Petersson J, Velcich A, Holm L, et al (2008) The inner of the two Muc2 mucin-dependent mucus layers in colon is devoid of bacteria. Proc Natl Acad Sci U S A 105: 15064–15069. 18806221 10.1073/pnas.0803124105 PMC2567493

[3] Johansson ME, Holmen Larsson JM, Hansson GC (2010) Microbes and Health Sackler Colloquium: The two mucus layers of colon are organized by the MUC2 mucin, whereas the outer layer is a legislator of host-microbial interactions. Proc Natl Acad Sci U S A 108 Suppl 14659–4665. 20615996 10.1073/pnas.1006451107 PMC3063600

[4] Johansson ME, Sjovall H, Hansson GC (2013) The gastrointestinal mucus system in health and disease. Nat Rev Gastroenterol Hepatol 10(6): 352–61. 23478383 10.1038/nrgastro.2013.35 PMC3758667

[5] Erdem A, Avelino F, Xicohtencatl-Cortes J, Girón J (2007) Host Protein Binding and Adhesive Properties of H6 and H7 Flagella of Attaching and Effacing Escherichia coli. J Bacteriol 189: 7426–7435. 17693516 10.1128/JB.00464-07 PMC2168434

[6] Linden SK, Florin TH, McGuckin MA (2008) Mucin dynamics in intestinal bacterial infection. PLoS One 3: e3952. 19088856 10.1371/journal.pone.0003952 PMC2601037

[7] Linden S, Mahdavi J, Hedenbro J, Boren T, Carlstedt I (2004) Effects of pH on Helicobacter pylori binding to human gastric mucins: identification of binding to non-MUC5AC mucins. Biochem J 384: 263–270. 15260802 10.1042/BJ20040402 PMC1134109

[8] McAuley JL, Linden SK, Png CW, King RM, Pennington HL, et al (2007) MUC1 cell surface mucin is a critical element of the mucosal barrier to infection. J Clin Invest 117: 2313–2324. 17641781 10.1172/JCI26705 PMC1913485

[9] Bergstrom KS, Kissoon-Singh V, Gibson DL, Ma C, Montero M, et al (2010) Muc2 protects against lethal infectious colitis by disassociating pathogenic and commensal bacteria from the colonic mucosa. PLoS Pathog 6: e1000902. 20485566 10.1371/journal.ppat.1000902 PMC2869315

[10] Maaser C, Housley MP, Iimura M, Smith JR, Vallance BA, et al (2004) Clearance of Citrobacter rodentium requires B cells but not secretory immunoglobulin A (IgA) or IgM antibodies. Infect Immun 72: 3315–3324. 15155635 10.1128/IAI.72.6.3315-3324.2004 PMC415672

[11] Kamada N, Kim YG, Sham HP, Vallance BA, Puente JL, et al (2012) Regulated virulence controls the ability of a pathogen to compete with the gut microbiota. Science 336: 1325–1329. 22582016 10.1126/science.1222195 PMC3439148

[12] Linden SK, Sutton P, Karlsson NG, Korolik V, McGuckin MA (2008) Mucins in the mucosal barrier to infection. Mucosal Immunol 1: 183–197. 19079178 10.1038/mi.2008.5 PMC7100821

[13] Verdugo P (1991) Mucin exocytosis. Am Rev Respir Dis 144: S33–37. 1892323 10.1164/ajrccm/144.3_pt_2.S33

[14] Ambort D, Johansson ME, Gustafsson JK, Nilsson HE, Ermund A, et al (2012) Calcium and pH-dependent packing and release of the gel-forming MUC2 mucin. Proc Natl Acad Sci U S A 109: 5645–5650. 22451922 10.1073/pnas.1120269109 PMC3326483

[15] Garcia MA, Yang N, Quinton PM (2009) Normal mouse intestinal mucus release requires cystic fibrosis transmembrane regulator-dependent bicarbonate secretion. J Clin Invest 119: 2613–2622. 19726884 10.1172/JCI38662 PMC2735925

[16] Joo NS, Krouse ME, Wu JV, Saenz Y, Jayaraman S, et al (2001) HCO3- transport in relation to mucus secretion from submucosal glands. JOP 2: 280–284. 11875272

[17] Gustafsson JK, Sjovall H, Hansson GC (2012) Ex vivo measurements of mucus secretion by colon explants. Methods Mol Biol 842: 237–243. 22259140 10.1007/978-1-61779-513-8_14

[18] Conlin VS, Wu X, Nguyen C, Dai C, Vallance BA, et al (2009) Vasoactive intestinal peptide ameliorates intestinal barrier disruption associated with Citrobacter rodentium-induced colitis. Am J Physiol Gastrointest Liver Physiol 297: G735–750. 19661153 10.1152/ajpgi.90551.2008

[19] Gustafsson JK, Ermund A, Ambort D, Johansson ME, Nilsson HE, et al (2012) Bicarbonate and functional CFTR channel are required for proper mucin secretion and link cystic fibrosis with its mucus phenotype. J Exp Med 209: 1263–1272. 22711878 10.1084/jem.20120562 PMC3405509

[20] Yu K, Lujan R, Marmorstein A, Gabriel S, Hartzell HC (2010) Bestrophin-2 mediates bicarbonate transport by goblet cells in mouse colon. J Clin Invest 120: 1722–1735. 20407206 10.1172/JCI41129 PMC2860923

[21] Greger R (2000) Role of CFTR in the colon. Annu Rev Physiol 62: 467–491. 10845099 10.1146/annurev.physiol.62.1.467

[22] Navabi N, McGuckin MA, Linden SK (2013) Gastrointestinal cell lines form polarized epithelia with an adherent mucus layer when cultured in semi-wet interfaces with mechanical stimulation. PLoS One 8: e68761. 23869232 10.1371/journal.pone.0068761 PMC3712011

[23] Johansson ME, Thomsson KA, Hansson GC (2009) Proteomic analyses of the two mucus layers of the colon barrier reveal that their main component, the Muc2 mucin, is strongly bound to the Fcgbp protein. J Proteome Res 8: 3549–3557. 19432394 10.1021/pr9002504

[24] Hasnain SZ, Evans CM, Roy M, Gallagher AL, Kindrachuk KN, et al (2011) Muc5ac: a critical component mediating the rejection of enteric nematodes. J Exp Med 208: 893–900. 21502330 10.1084/jem.20102057 PMC3092342

[25] Khan WI, Abe T, Ishikawa N, Nawa Y, Yoshimura K (1995) Reduced amount of intestinal mucus by treatment with anti-CD4 antibody interferes with the spontaneous cure of Nippostrongylus brasiliensis-infection in mice. Parasite Immunol 17: 485–491. 8552418 10.1111/j.1365-3024.1995.tb00919.x

[26] Ishikawa N, Wakelin D, Mahida YR (1997) Role of T helper 2 cells in intestinal goblet cell hyperplasia in mice infected with Trichinella spiralis. Gastroenterology 113: 542–549. 9247474 10.1053/gast.1997.v113.pm9247474

[27] Shiomi H, Masuda A, Nishiumi S, Nishida M, Takagawa T, et al (2010) Gamma interferon produced by antigen-specific CD4+ T cells regulates the mucosal immune responses to Citrobacter rodentium infection. Infect Immun 78: 2653–2666. 20351140 10.1128/IAI.01343-09 PMC2876554

[28] Malmberg EK, Noaksson KA, Phillipson M, Johansson ME, Hinojosa-Kurtzberg M, et al (2006) Increased levels of mucins in the cystic fibrosis mouse small intestine, and modulator effects of the Muc1 mucin expression. Am J Physiol Gastrointest Liver Physiol 291: G203–210. 16500918 10.1152/ajpgi.00491.2005

[29] Parmley RR, Gendler SJ (1998) Cystic fibrosis mice lacking Muc1 have reduced amounts of intestinal mucus. J Clin Invest 102: 1798–1806. 9819365 10.1172/JCI3820 PMC509129

[30] Tam PY, Verdugo P (1981) Control of mucus hydration as a Donnan equilibrium process. Nature 292: 340–342. 7195985 10.1038/292340a0

[31] Verdugo P (1991) Mucin exocytosis. 144: S33–S37. 10.1164/ajrccm/144.3_pt_2.S33 1892323

[32] Stefanovic B, Stefanovic L, Schnabl B, Bataller R, Brenner DA (2004) TRAM2 protein interacts with endoplasmic reticulum Ca2+ pump Serca2b and is necessary for collagen type I synthesis. Mol Cell Biol 24: 1758–1768. 14749390 10.1128/MCB.24.4.1758-1768.2004 PMC344171

[33] Muchekehu RW, Quinton PM (2010) A new role for bicarbonate secretion in cervico-uterine mucus release. J Physiol 588: 2329–2342. 20478977 10.1113/jphysiol.2010.187237 PMC2915510

[34] Skinn AC, Vergnolle N, Zamuner SR, Wallace JL, Cellars L, et al (2006) Citrobacter rodentium infection causes iNOS-independent intestinal epithelial dysfunction in mice. Can J Physiol Pharmacol 84: 1301–1312. 17487239 10.1139/y06-086

[35] Umar S, Scott J, Sellin JH, Dubinsky WP, Morris AP (2000) Murine colonic mucosa hyperproliferation. I. Elevated CFTR expression and enhanced cAMP-dependent Cl(-) secretion. Am J Physiol Gastrointest Liver Physiol 278: G753–764. 10801268 10.1152/ajpgi.2000.278.5.G753

[36] Golin-Bisello F, Bradbury N, Ameen N (2005) STa and cGMP stimulate CFTR translocation to the surface of villus enterocytes in rat jejunum and is regulated by protein kinase G. Am J Physiol Cell Physiol. 289: C708–716. 10.1152/ajpcell.00544.2004 15872007

[37] Sugi K, Musch MW, Field M, Chang EB (2001) Inhibition of Na+,K+-ATPase by interferon gamma down-regulates intestinal epithelial transport and barrier function. Gastroenterology 120: 1393–1403. 11313309 10.1053/gast.2001.24045

[38] Tan J, Qiao W, Wang J, Xu F, Li Y, et al (2008) IFP35 is involved in the antiviral function of interferon by association with the viral tas transactivator of bovine foamy virus. J Virol 82: 4275–4283. 18305040 10.1128/JVI.02249-07 PMC2293045

[39] Bergstrom KS, Guttman JA, Rumi M, Ma C, Bouzari S, et al (2008) Modulation of intestinal goblet cell function during infection by an attaching and effacing bacterial pathogen. Infect Immun 76: 796–811. 17984203 10.1128/IAI.00093-07 PMC2223480

[40] Specian RD, Neutra MR (1980) Mechanism of rapid mucus secretion in goblet cells stimulated by acetylcholine. J Cell Biol 85: 626–640. 7391135 10.1083/jcb.85.3.626 PMC2111470

[41] Philips MA, Vikesa J, Luuk H, Jonson L, Lillevali K, et al (2009) Characterization of MYG1 gene and protein: subcellular distribution and function. Biol Cell 101: 361–373. 19014353 10.1042/BC20080086

[42] Schweinfest CW, Spyropoulos DD, Henderson KW, Kim JH, Chapman JM, et al (2006) slc26a3 (dra)-deficient mice display chloride-losing diarrhea, enhanced colonic proliferation, and distinct up-regulation of ion transporters in the colon. J Biol Chem 281: 37962–37971. 17001077 10.1074/jbc.M607527200

[43] Sterling D, Brown NJ, Supuran CT, Casey JR (2002) The functional and physical relationship between the DRA bicarbonate transporter and carbonic anhydrase II. Am J Physiol Cell Physiol 283: C1522–1529. 12372813 10.1152/ajpcell.00115.2002

[44] Vidyasagar S, Rajendran VM, Binder HJ (2004) Three distinct mechanisms of HCO3- secretion in rat distal colon. Am J Physiol Cell Physiol 287: C612–621. 15308466 10.1152/ajpcell.00474.2003

[45] Singh AK, Xia W, Riederer B, Juric M, Li J, et al (2013) Essential role of the electroneutral Na+-HCO3- cotransporter NBCn1 in murine duodenal acid-base balance and colonic mucus layer build-up in vivo. J Physiol 591: 2189–2204. 23401617 10.1113/jphysiol.2012.247874 PMC3634528

[46] Navabi N, Johansson ME, Raghavan S, Linden SK (2013) Helicobacter pylori infection impairs the mucin production rate and turnover in the murine gastric mucosa. Infect Immun 81: 829–837. 23275091 10.1128/IAI.01000-12 PMC3584886

[47] Gustafsson JK, Ermund A, Johansson ME, Schutte A, Hansson GC, et al (2012) An ex vivo method for studying mucus formation, properties, and thickness in human colonic biopsies and mouse small and large intestinal explants. Am J Physiol Gastrointest Liver Physiol 302: G430–438. 22159279 10.1152/ajpgi.00405.2011 PMC4073982

[48] Amann RI, Binder BJ, Olson RJ, Chisholm SW, Devereux R, et al (1990) Combination of 16S rRNA-targeted oligonucleotide probes with flow cytometry for analyzing mixed microbial populations. Applied and environmental microbiology 56: 1919–1925. 2200342 10.1128/aem.56.6.1919-1925.1990 PMC184531

[49] Wisniewski JR, Zougman A, Nagaraj N, Mann M (2009) Universal sample preparation method for proteome analysis. Nat Methods 6: 359–362. 19377485 10.1038/nmeth.1322

[50] Rodriguez-Pineiro AM, Post SV, Johansson ME, Thomsson KA, Nesvizhskii AI, et al (2012) Proteomic Study of the Mucin Granulae in an Intestinal Goblet Cell Model. J Proteome Res 11(3): 1879–90. 22248381 10.1021/pr2010988 PMC3292267

[51] Cox J, Mann M (2008) MaxQuant enables high peptide identification rates, individualized p.p.b.-range mass accuracies and proteome-wide protein quantification. Nat Biotechnol 26: 1367–1372. 19029910 10.1038/nbt.1511

[52] Cox J, Neuhauser N, Michalski A, Scheltema RA, Olsen JV, et al (2011) Andromeda: a peptide search engine integrated into the MaxQuant environment. J Proteome Res 10: 1794–1805. 21254760 10.1021/pr101065j

[53] Conesa A, Gotz S, Garcia-Gomez JM, Terol J, Talon M, et al (2005) Blast2GO: a universal tool for annotation, visualization and analysis in functional genomics research. Bioinformatics 21: 3674–3676. 16081474 10.1093/bioinformatics/bti610

