# Supplementary material for: Dynamic Changes in Mucus Thickness and Ion Secretion during Citrobacter rodentium Infection and Clearance
Source: PLoS One. 2013 Dec 30;8(12):e84430. doi: 10.1371/journal.pone.0084430 (PMC3875541; doi:10.1371/journal.pone.0084430)
Supplement: Table S1 — Proteins found predominantly in the mucus of infected mice at day 14 (at least in 3/4 infected animals and in none or only 1/4 control mice), or almost absent from them (present in at least 3/4 control mice, but in none or only 1/4 infected mice). (DOCX) [file pone.0084430.s001.docx]

| **PROTEINS PREDOMINANT IN MUCUS OF INFECTED MICE** | | |  |  | |
| --- | --- | --- | --- | --- | --- |
| **UniProt ID** | **Gene Name** | **Protein Name** | | | |
| Q9D2R0 | *Aacs* | Acetoacetyl-CoA synthetase | | | |
| Q07417 | *Acads* | Butyryl-CoA dehydrogenase | | | |
| O35643 | *Adtb1* | Adapter-related protein complex 1 subunit beta-1 | | | |
| P24822 | *Akp3* | Alkaline phosphatase 3 | | | |
| O55143 | *Atp2a2* | Calcium pump 2 | | | |
| Q9CPQ8 | *Atp5l* | ATP synthase subunit g, mitochondrial | | | |
| Q8VE37 | *Chc1* | Chromosome condensation protein 1 | | | |
| Q8BMK4 | *Ckap4* | Cytoskeleton-associated protein 4 | | | |
| Q62167 | *D1Pas1-rs2* | D1Pas1-related sequence 2 | | | |
| Q9JII5 | *Dazap1* | Deleted in azoospermia-associated protein 1 | | | |
| Q91VR5 | *Ddx1* | DEAD box protein 1 | | | |
| Q9JIK5 | *Ddx21* | DEAD box protein 21 | | | |
| Q8BVI4 | *Dhpr* | Dihydropteridine reductase | | | |
| P48024 | *Eif1* | Eukaryotic translation initiation factor 1 | | | |
| Q64520 | *Gmk* | GMP kinase | | | |
| P38060 | *Hmgcl* | 3-hydroxy-3-methylglutarate-CoA lyase | | | |
| Q9D8C4 | *Ifi35* | Interferon-induced 35 kDa protein homolog | | | |
| Q3UQ44 | *Iqgap2* | Ras GTPase-activating-like protein IQGAP2 | | | |
| P46978 | *Itm1* | Integral membrane protein 1 | | | |
| P54726 | *Mhr23a* | UV excision repair protein RAD23 homolog A | | | |
| P62960 | *Msy1* | CCAAT-binding transcription factor I subunit A | | | |
| Q9DCJ5 | *Ndufa8* | NADH dehydrogenase [ubiquinone] 1 alpha subcomplex subunit 8 | | | |
| P11031 | *Pc4* | Positive cofactor 4 | | | |
| P47955 | *Rplp1* | 60S acidic ribosomal protein P1 | | | |
| Q64213 | *Sf1* | Splicing factor 1 | | | |
| Q8BMA6 | *Srp68* | Signal recognition particle 68 kDa protein | | | |
| Q64356 | *Svp6* | Seminal vesicle secretory protein 6 | | | |
| Q921F2 | *Tardbp* | TAR DNA-binding protein 43 | | | |
| P39447 | *Tjp1* | Tight junction protein 1 | | | |
|  |  |  | | | |
| **PROTEINS PREDOMINANT IN MUCUS OF CONTROL MICE** | | |  | |  |
| **Protein ID** | **Gene Names** | **Protein Names** | | | |
| Q9D0A3 | - | UPF0552 protein C15orf38 homolog | | | |
| Q7TMS5 | *Abcg2* | ATP-binding cassette sub-family G member 2 | | | |
| Q91VR2 | *Atp5c1* | F-ATPase gamma subunit | | | |
| Q91XV3 | *Basp1* | Brain acid soluble protein 1 | | | |
| O89110 | *Casp8* | Caspase-8 | | | |
| Q9JIF7 | *Copb* | Coatomer subunit beta | | | |
| O55029 | *Copb2* | Coatomer subunit beta | | | |
| Q91VC7 | *Cpi17* | Protein kinase C-potentiated inhibitor protein of 17 kDa | | | |
| Q8BIK4 | *D14Wsu89e* | Dedicator of cytokinesis protein 9 | | | |
| Q61160 | *Fadd* | FAS-associated death domain protein | | | |
| P09528 | *Fth* | Ferritin heavy chain | | | |
| Q99KB8 | *Glo2* | Glyoxalase II | | | |
| Q5EBG6 | *Gm479* | Heat shock protein beta-6 | | | |
| P17156 | *Hcp70.2* | Heat shock-related 70 kDa protein 2 | | | |
| Q00PI9 | *Hnrnpul2* | Heterogeneous nuclear ribonucleoprotein U-like protein 2 | | | |
| Q3UV17 | *Krt76* | Keratin-76 | | | |
| Q60692 | *Lmp19* | Low molecular mass protein 19 | | | |
| P26645 | *Macs* | Myristoylated alanine-rich C-kinase substrate | | | |
| Q9DCS3 | *Mecr* | Trans-2-enoyl-CoA reductase, mitochondrial | | | |
| P28825 | *Mep1a* | Meprin A subunit alpha | | | |
| Q8VE97 | *MNCb-2616* | Splicing factor, arginine/serine-rich 4 | | | |
| Q99JI1 | *Mustn1* | Musculoskeletal embryonic nuclear protein 1 | | | |
| Q9JK81 | *Myg1* | Protein Gamm1 | | | |
| Q9D6J6 | *Ndufv2* | NADH dehydrogenase [ubiquinone] flavoprotein 2, mitochondrial | | | |
| Q61036 | *Pak3* | Serine/threonine-protein kinase PAK 3 | | | |
| Q05920 | *Pc* | Pyruvic carboxylase | | | |
| Q6W8Q3 | *Pcp4l1* | Purkinje cell protein 4-like protein 1 | | | |
| O70209 | *Pdlim3* | PDZ and LIM domain protein 3 | | | |
| Q9CR16 | *Ppid* | Peptidyl-prolyl cis-trans isomerase D | | | |
| Q9D939 | *Sult1c1* | Sulfotransferase 1C2 | | | |
| Q7TQD2 | *Tppp* | Tubulin polymerization-promoting protein | | | |
| Q7TMM9 | *Tubb2* | Tubulin beta-2 | | | |
| Q9R0P9 | *Uchl1* | Ubiquitin carboxyl-terminal hydrolase isozyme L1 | | | |
